# Emotion-related impulsivity and suicidal ideation and behavior in schizophrenia spectrum disorder: a pilot fMRI study

**DOI:** 10.3389/fpsyt.2024.1408083

**Published:** 2024-06-26

**Authors:** Matthew J. Hoptman, Kathryn T. Evans, Zamfira Parincu, Allison M. Sparpana, Elizabeth F. Sullivan, Anthony O. Ahmed, Dan V. Iosifescu

**Affiliations:** ^1^ Division of Clinical Research, Nathan S. Kline Institute for Psychiatric Research, Orangeburg, NY, United States; ^2^ Department of Psychiatry, NYU Grossman School of Medicine, New York, NY, United States; ^3^ Department of Psychiatry, Weill Cornell Medicine, White Plains, NY, United States

**Keywords:** suicidal ideation and behavior, schizophrenia spectrum disorder, emotion regulation, fMRI, urgency

## Abstract

**Introduction:**

Suicidal ideation and behavior (SIB) are serious problems in people with schizophrenia spectrum disorders (SSD). Nevertheless, relatively little is known about the circuitry underlying SIB in SSD. Recently, we showed that elevated emotional impulsivity (urgency) was associated with SIB in SSD. Here we examined brain activity in people with SSD and elevated SIB.

**Methods:**

We tested 16 people with SSD who had low SIB and 14 people with high SIB on a task in which emotion regulation in response to affective pictures was implicitly manipulated using spoken sentences. Thus, there were neutral pictures preceded by neutral statements (NeutNeut condition), as well as negative pictures preceded by either negative (NegNeg) or neutral (NeutNeg) statements. After each picture, participants rated how unpleasant each picture was for them. The latter two conditions were compared to the NeutNeut condition. We compared the emotion-regulated condition (NeutNeg) to the unregulated condition (NeutNeut). Statistics were threshold using threshold free cluster enhancement (TFCE).

**Results:**

People in the low SIB group showed higher activation in this contrast in medial frontal gyrus, right rostral anterior cingulate, bilateral superior frontal gyrus/DLPFC, and right middle cingulate gyrus, as well as right superior temporal gyrus.

**Discussion:**

This study provides clues to the neural basis of SIB in SSD as well as underlying mechanisms.

## Introduction

Suicidal ideation and behavior (SIB) are substantially elevated in schizophrenia spectrum disorders (SSD), with up to 40% of people with SSD having a lifetime suicide attempt (SA; (1, 2) and 5–6% dying by suicide (3–5), but that rate may be as high as 10% (6). This rate is at least as high as in major depressive disorder (7). Our recent work suggests that emotion-related impulsivity (urgency) is an important determinant of SIB, at least in SSD (8).

Urgency refers to rash action in the context of strong emotions and is measured using the Urgency, (Lack of) Premeditation, (Lack of) Perseverance, and Sensation Seeking Scale [UPPS; (9)]. Urgency has been further divided into positive urgency (PU; positive emotions) and negative urgency (NU; negative emotions), leading to the development of the UPPS-P (10). In a meta-analysis (11), urgency was found to be the impulsivity dimension most correlated with psychiatric conditions characterized by high levels of SIB, including alcohol/substance use disorders (12). Urgency may underlie (13) a recently identified superordinate general psychopathology (“*p*”) factor that may explain psychiatric disorders better than the traditional 3 factor model of internalizing, externalizing, and thought disorders (14).

We have extended these findings to SSD (8, 15). Earlier studies of impulsivity and aggression in SSD offered mixed results (16–18), but none examined urgency, as emotion had been explicitly excluded from some of the more commonly used measures, such as the Barratt Impulsiveness Scale (19). We found that NU and PU were selectively elevated in SSD with large effect sizes (*d* > 1.2; (15) and were related to self-reported aggression.

Recently, we found that SI is strongly correlated with NU in SSD, even more strongly than with depressive symptoms (8). We also found that NU completely mediated the relationship between depressive symptoms and SI, suggesting NU may be a pathway linking depression to SIB in SSD. Moreover, NU correlated with lifetime suicide attempts (*r_s_* = 0.48, *p* = 0.003).

Several MRI studies have examined SIB in SSD, most with small sample sizes. Structural MRI studies report lower gray matter density in left orbitofrontal (OFC) and superior temporal cortices in SSD attempters compared to nonattempters (20), higher bilateral volume of inferior frontal white matter in SSD attempters compared to psychiatric and healthy controls (21), and larger amygdala volumes, which was also correlated with self-directed aggression, in suicide attempters compared to psychiatric and healthy controls (22). Attempters with SSD have also been found to have lower cortical thickness in right dorsolateral prefrontal cortex (DLPFC) and superior temporal cortex compared with non-suicidal patients (23). The largest structural imaging study on SIB in psychoses (24) found lower gray matter volume in bilateral superior and middle frontal cortex, inferior and superior temporal cortex, left superior parietal regions, and right insula and thalamus. These preliminary findings suggest morphological differences in cognitive control and affective processing related to SIB.

SIB in SSD has also been evaluated using fMRI, implicating some of the same brain regions. In a study of patients with self-harm history, patients without self-harm histories, and controls (25), those with a history of self-harm showed activation intermediate to that of the other two groups (controls were highest) in right DLPFC and left ventral posterior cingulate during the no-go condition of a go/no-go task. Activation in right DLPFC correlated with severity of suicidal ideation in the self-harm group. In subjects with recent-onset SSD, Minzenberg et al. (26) found that those with past suicidal ideation had higher functional connectivity (FC) between the dorsal anterior cingulate cortex (dACC) and the precuneus during conflict monitoring. Moreover, the intensity of suicidal ideation at its worst point was associated with higher FC between the dACC and both the medial parietal lobe and striatothalamic nuclei. Past suicidal behavior was associated with reduced dACC FC in lateral and medial PFC, parietal, and temporal cortical areas. Although these studies identified the cortical changes associated with SIB in SSD patients, we still do not understand the role of urgency in SIB and the neurobiological pathways underlying these relationships.

As initially proposed, NU was posited to involve emotionally relevant circuitry, including the OFC and ventromedial prefrontal cortex (vmPFC), as well as the amygdala (10). Studies have since confirmed and expanded on regions implicated in this circuitry. In a resting state fMRI study, urgency (mean of NU and PU) was positively related to the amplitude of low frequency fluctuations in the lateral OFC, vmPFC, right DLPFC, left inferior frontal gyrus, and middle frontal gyrus, and posterior cingulate cortex/precuneus in healthy volunteers (27). In social drinkers, NU mediated the relationship between amygdala and right OFC activation in response to negative emotion pictures as well as general risk-taking (28). Recently, in a transdiagnostic sample, Elliott et al. (29) found that the local gyrification index (a ratio of the amount of cortex buried in sulci to the amount visible on the surface) of lateral OFC correlated with “feelings trigger actions,” a measure highly similar to urgency.

We previously found that urgency is correlated with reduced cortical thickness in ventral prefrontal and limbic regions, including the rostral anterior cingulate (rACC) and frontal pole, as well as lateral and medial OFC in SSD (15). Moreover, we found that higher urgency correlates with lower resting state FC in these regions.

Urgency has been related to dysfunctional emotion regulation (ER), which includes the explicit strategies used to control one’s experienced emotions (30). People with SSD have substantial deficits in ER (31, 32). Moreover, NU and maladaptive ER strategies are positively correlated (33). Finally, a number of the regions implicated in urgency are also associated with emotion regulation (34, 35). There may also be neurobiological correlates between urgency and ER, specifically among frontotemporal and limbic regions.

The development of paradigm measures of urgency is in its infancy and has had mixed success (36, 37); there are no validated fMRI tasks for NU in SSD. Here, we used an emotion regulation task in which participants performed implicit reappraisal of affective pictures based on spoken sentences that preceded those images (38, 39). Based on the literature, we predicted that people in the high SIB group would show lower activation on the task compared to those with low SIB in regions related to emotion regulation. We also determined whether activation on this task was associated with NU.

## Material and methods

Thirty-five patients with SSDs from our prior work (8) provided clinical data for the current study. Inpatient and outpatient participants were recruited from Rockland Psychiatric Center and referred by other researchers. Of these, we had a final imaging sample of 30 participants: two of the participants could not hear the spoken statements, one participant could not tolerate scanning, one participant showed excessive data loss (>45% censored volumes, see below) and one showed low temporal signal to noise ratio (tSNR) = 59.5, which was 3.84.SDs below the group mean (168.88 ± 28.52).

The Columbia Suicide Severity Rating Scale (40) entails a semi structured interview and review of medical records to assess past year and lifetime SIB and was used to form two groups. The low SIB group (*n* = 16) was comprised of people with scores of 1 or less (lifetime) on the C-SSRS and with no lifetime suicide attempts. The high SIB group (*n* = 14) consisted of people with scores of 3+ in the past 12 months and/or at least two lifetime suicide attempts. This measure also provided data on suicide attempts. The final neuroimaging sample was 30, of whom 16 were in the low SIB group and 14 were in the high SIB group.

DSM-5 diagnosis was confirmed by either the Mini International Neuropsychiatric Interview, v. 7.0.2 (41) or the Structural Clinical Interview for DSM-5 (42). Twenty-three participants had schizophrenia and 7 had schizoaffective disorder. Medication dosages were converted into chlorpromazine (CPZ) equivalents (43). We also compared the number of people taking first generation antipsychotics, second generation antipsychotics, or both. People with recent (past 3 months) substance use disorders were excluded from the study. All procedures were approved by the Nathan Kline Institute Institutional Review Board and all participants signed informed written consent.

### Measures

The Beck Scale for Suicidal Ideation [BSSI; (44)] is a 21-item questionnaire that asks participants about past-week suicidal ideation and attempts. The first 19 items provided a score for suicidal ideation.

We administered the UPPS-P scale (9, 10) that measures NU and PU. We have extensive experience using this scale in SSD.

Positive and Negative Syndrome Scale [PANSS; (45)] provides data on positive, negative, depression, cognitive, and excitement factors (46) and was used to assess past-week psychopathology. We also computed the Excited Component [PANSS-EC; (47)].

The participants described above were tested on an fMRI task which implicitly manipulated emotion regulation in response to affective pictures using neutral and negative sentences preceded by spoken statements (38, 39). The task was programmed in E-prime, v. 2.0 (Psychology Software Tools, Pittsburgh, PA) and was rear-projected to participants in the scanner.

### MRI acquisition

Scanning took place at NKI’s Center for Biomedical Imaging and Neuromodulation (CBIN) using a Siemens 3T TiM Trio and a 32-channel head coil. We collected an anatomical scan (MPRAGE) to provide cortical thickness measurements and to allow intersubject registration of functional images. Sequence parameters are provided in **Table 1**.

**Table 1:** Parameters of sequences used.

Scan	TR/TE (ms)	TI (ms)	Matrix	FOV^1^	Slices	Thick^1^	GRAPPA	MB^2^
MPRAGE	2530/2.3	1340	256^2^	250	176	1	2	–
Resting/task	1400/30	–	90^2^	180	64	2	2	4

^1^Thickness and FOV in mm; ^2^MB, multiband factor.

### fMRI task

Participants viewed full-frame negative and neutral pictures from the International Affective Pictures System (IAPS; (48)) presented for 3 seconds each. Neutral pictures were preceded by a spoken neutral statement (NeutNeut), whereas negative pictures were preceded by either negative (NegNeg) or neutral (NeutNeg) spoken statements. These sentences were presented over a 7-second window. The participant then rated the unpleasantness of the picture using the Self-Assessment Manikin (49); up to 5 seconds, self-paced). Between the picture offset and the rating screen, there was a variable delay of 0.5 - 1.5 seconds. There were 22 pictures in each condition; the task was presented in two blocks of approximately 9 min (66 trials total) with equal numbers of trials for each condition in each block. The order of trial presentation was randomized for each participant. To avoid effects of sedation, study assessments were not done within 12 hours of PRN medication administration.

### Image processing

fMRI data were preprocessed using AFNI (50). We used AFNI’s *afni_proc.py* metascript to perform motion correction, registration of images into standard space, and spatial smoothing (4mm FWHM Gaussian kernel), as well as regression of nuisance covariates (12 motion parameters). Finally, data were scaled to percent signal change (PSC). The regression of nuisance parameters was done in the deconvolution step. Next, we created stimulus onset regressors (convolved with a BLOCK function) for each condition (NeutNeut, NeutNeg, NegNeg) at time of sentence onset and time of picture onset, to construct a general linear model (GLM) based on the PSC images. We also created a regressor for the onset of the rating screen using a gamma variate function. Image frames with more than 0.9mm of motion with respect to the prior frame were censored. Motion parameters and their derivatives were nuisance regressors, as were rating onset times. Analyses were limited by a mask in which 70% of participants had data. We computed a contrast between NeutNeg- conditions for the time of picture onset. Data were thresholded at *p* < 0.05, corrected, using threshold-free cluster enhancement (51).

## Results

Demographic data are shown in **Table 2**. SIB Groups did not differ in age, sex, education, or medication dosages.

**Table 2:** Demographic variables in people in the high and low SIB groups.

Variables	Low SIB Group	High SIB Group	Statistic		*d/η^2^/w*
	M (SD)	M(SD)	*t/U/*/χ^2^	*p*	
**Age (years)**	40.1 (11.0)	42.1 (10.3)	-0.50	.62	-0.18
**Sex (M/F)**	14/2	12/2	0.02	.65	0.02
**Education (years)^1^**	3.9 (1.6)	4.9 (1.4)	-1.66	.11	-0.66
**Handness (RH/LH)^2^**	14/2	11/2	0.05	0.82	0.04
**CPZ Equivalents^3^ (mg)**	936.4 (1017.3)	1576.9 (764.7)	-1.42	.17	-0.71
**C-SSRS SI (life)**	0 [0,1]	5 [4,5]	176**^4^**	<.001**	0.90
**C-SSRS Attempts (life)**	0 [0,0]	3 [1,8]	224^4^	<.001**	0.88
**C-SSRS SI (past year)**	0.12 (0.34)	1.36 (1.28)	-3.50	.003**	-1.32
**C-SSRS Attempts (past year)**	0 [0,0]	0 [0,0]	112^4^	1.00	0.00
**BSSI Suicidal Ideation**	0 [0,8]	4 [0,14]	175.5^4^	.007**	0.34
**Negative Urgency (total)**	23.0 (8.0)	31.8 (7.4)	-3.12	.004**	-1.14
**Positive Urgency (total)**	26.5 (10.1)	30.1 (10.8)	-0.95	.20	-0.34
**PANSS Negative Scale**	17.8 (8.6)	14.4 (4.8)	1.20	.35	0.49
**PANSS Excitement Scale**	8 [6,19]	11 [6,17]	162.5**^4^**	.034	0.16
**PANSS Cognitive Scale**	13.5 [7,21]	12.5 [5,17]	81.5**^4^**	.21	0.06
**PANSS Positive Scale**	10.9 (4.9)	13.3 (4.7)	-1.37	.18	-0.50
**PANSS Depression Scale**	9.8 (4.7)	14.8 (4.4)	-3.00	.006**	-1.10

CPZ equivalents (43); FG, first generation antipsychotic; SG, second generation antipsychotic; C-SSRS, Columbia-Suicide Severity Rating Scale (40); LH, Left-handed; RH, right-handed; BSSI, Beck Scale for Suicidal Ideation (52); PANSS, Positive and Negative Syndrome Scale (45, 53); *Significant at p <.05; **Significant at p<.01. ^1^Missing for 14 subjects; ^2^Missing for 11 subjects; ^3^Missing for 11 subjects; ^4^Mann-Whitney U test; descriptive statistics presented for each group are median and [minimum, maximum].

The behavioral dependent measure was the difference in unpleasantness ratings for NegNeg vs. NeutNeg trials. We would expect unpleasantness ratings to be higher for NegNeg because of the buffering effect of the neutral statements. Ratings were lower for NeutNeg than NegNeg trials [*t*(28) = -5.28, *p* < 1.3 x 10^-5^] suggesting that participants could perform the task. Thus, the task worked as designed. Ratings did not differ between groups (|*t*| < 1.22, *p*s <.23).

**Figure 1:**
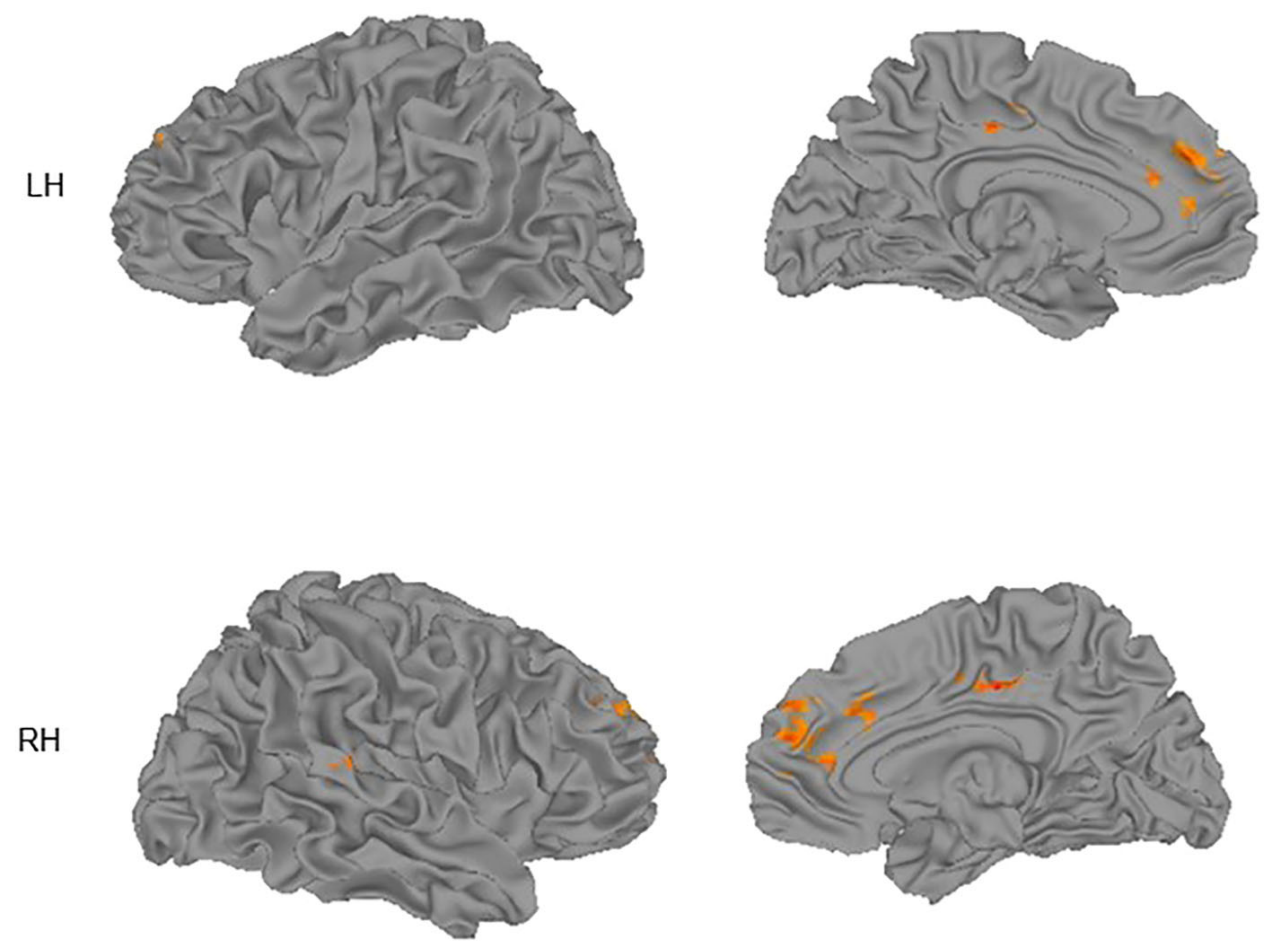
Group differences (low SIB group > high SIB group) in activation for the Neutral/Negative – Neutral/Neutral contrast superimposed on cortical surface maps. Surfaces shown are lateral (left column) and medial (right column) for left (LH) and right (RH) hemispheres. Images thresholded using nonparametric threshold-free cluster enhancement (TFCE).

Participants in the high SIB group had lower activation in the right anterior cingulate cortex/BA 32 [MNI coordinate = (11, 35, 20)] compared to the low SIB group. Urgency-relevant circuitry also was activated in the NeutNeg > NeutNeut contrast between groups (**Figure 1**), demonstrating reappraisal-related activation in the left superior medial frontal gyrus and superior frontal gyrus (SFG) as well as right middle cingulate, superior frontal gyrus, superior temporal gyrus, and DLPFC. Within-group analyses show that people in the high SIB group showed deactivation in these regions, whereas those in the low SIB group showed a weaker pattern of elevated activation in these regions at a more liberal threshold of p = .005 and cluster size of 9, because of the smaller group sizes (see **Supplementary Figures 1**, **2**). Consistent with recent studies (15, 27), activation in right middle cingulate, right superior temporal gyrus, and right DLPFC correlated significantly and negatively with NU (*r_s_* < –0.37, *p* < 0.042). A voxelwise analysis of these correlations was inconclusive, possibly owing to the low power to detect correlations (**Supplementary Figure 3**).

## Discussion

In this study, we showed that compared to those with SSD and low levels of SIB, people with SSD and high levels of SIB had lower BOLD activation in several regions on an emotion regulation task. These regions included medial frontal gyrus, right rACC, bilateral superior frontal gyrus/DLPFC, and right middle cingulate gyrus. We also found that activation in right middle cingulate, right superior temporal gyrus, and right DLPFC were significantly and negatively correlated with negative urgency. This adds to the growing literature on the neural circuitry underlying NU.

Although the present task has been viewed as tapping implicit emotional reappraisal (39), rather than urgency, it may nonetheless be a good urgency proxy task because 1) it evokes strong negative affect that can lead to reflexive emotional regulatory responses (33), 2) these responses are correlated with NU (33), 3) many of the brain activation deficits seen in the high SIB group correlate with urgency, and 4) it has no floor effects. At present, there is only one cognitive task (an emotional stop signal task) that has been validated against negative urgency measures (36); however, it has not been tested in SSD.

Negative urgency is elevated in SSD (15), and this elevation is related to SIB, even after accounting for depressive symptoms (8). Thus, the current study extends our prior results regarding violence in schizophrenia and are consistent with other studies of the circuitry associated with urgency in other disorders. The regions that showed activation differences are also implicated in emotion regulation (34, 35). The ability to regulate one’s emotions effectively plays a key role in SIB and has been established as a known deficiency in SSD (31, 54–56). Interestingly, the low SIB group showed positive activation in these regions, whereas the high SIB group showed negative activation (i.e., deactivation). This suggests that the relevant circuitry is highly disrupted in the high SIB group in a manner that could predict SIB.

The NegNeg and NegNeg vs. NegNeut analyses did not show significant differences, despite differences showing up on the behavioral measure. It is possible that the range of activation on these contrasts were restricted given that both NegNeg and NeutNeg contained negatively valanced pictures as compared to the NeutNeut condition. Disentangling these relationships will be the subject of future research.

As can be seen in **Figure 1**, activation in midline regions is represented on both hemispheres. This is the case because in **Table 3** we report peak locations. However, the midline clusters we detected extended into the other hemisphere, suggesting that both left and right midline regions contribute to the observed activation.

**Table 3:** Clusters showing significant differences in activation between low and high SIB groups.

Region	Talairach Coordinates (mm)	Size (voxels)	*t*
**L Medial frontal gyrus/BA9**	-1, 45, 24	375	4.76
**R Rostral anterior Cingulate/BA32**	11, 35, 12	154	4.22
**R Middle cingulate/BA24**	3, -21, 38	94	5.66
**R Superior temporal gyrus**	57, -35, 14	23	4.48
**L Superior frontal gyrus**	-13, 51, 32	12	4.08
**R Superior frontal gyrus/** **middle frontal gyrus (DLPFC)**	29, 39, 30	11	3.84
**R Superior frontal gyrus/BA10**	27, 57, 14	9	4.31
**R Superior frontal gyrus/BA9**	25, 47, 30	9	3.73

Existing literature (e.g., 57) has established disturbances in frontal-cortical regions and their interacting networks, which may present clinically as disturbances in the control of emotions and behavior, occasionally presenting as SIB in those with SSDs. Consistent with the findings in the present study emphasizing the role of lower activation in the medial frontal gyrus in our high SIB group, Minzenberg et al.’s (57) study found an inverse relationship between suicidal ideation and activation in this region as well as in the left rostral pole and the right dorsal anterior cingulate gyrus. In SSD patients, the lower activation, specifically in the medial frontal gyrus and other frontal-cortical regions, has been linked to impaired goal representation, which was associated with increased suicidal ideation. The medial frontal gyrus has also been associated with several cognitive processes associated with emotion regulation and urgency, including decision making, reasoning, and discrimination (58), suggesting that disruptions in this region could play a key role in the dysregulation experienced in SSD populations with high SIB. As previously discussed, this dysregulation and inability to apply traditional coping mechanisms has been shown to lead to increased rates of urgency, particularly NU. The current findings are consistent with our prior findings that urgency is correlated with lower right frontal pole thickness in SSD (15). These frontal cortical regions are therefore important regions of interest for continuing research focusing on pathologies stemming from emotion dysregulation and urgency.

Additionally, we found decreased activation in the right rACC cortex in individuals with SSD and high levels of SIB. In previous studies, we found that urgency was correlated with reduced cortical thickness in the rACC in SSD. This finding is novel, as we are aware of no previous research establishing the link among rACC, suicidality, and SSD. Our results converge, however, with findings that rACC cortex dysfunction is tied to treatment response in major depressive disorder (MDD), with higher regional activity being associated with better treatment reactivity (59). In several past studies, long-term treatment resistance in MDD was found to be associated with increased hopelessness and suicidal ideation (60, 61). Differences in anterior cingulate cortex with SSD have been noted both anatomically and physiologically (62). Our data suggest that hypoactivation in the region is associated with poor emotion regulation, possibly through urgency.

The present study’s finding of decreased BOLD activation in the DLPFC in SSD individuals with high SIB compared to low SIB is consistent with the results of prior work (23) and fits into past literature implicating the region in urgency (27). Moreover, we found that lower DLPFC activation was associated with higher levels of negative urgency. Past studies have shown that cortical thinning in the DLPFC may impact fronto-thalamic functioning, which could affect both cognitive and emotional control processes (23). These kinds of disturbances have been shown to lead to suicidal behavior in MDD through the disinhibition of emotional responses, and a reduced ability to adapt to stressful behavior (63). These kinds of responses are formally similar to urgency, but Jia et al. (63) did not examine urgency in their study. Although the findings in this latter study do not extend to patients with SSD in the current work, similar differences found in the superior frontal gyrus have been reported, not just in MDD, but in suicide attempters with SSD as well as other psychiatric disorders (64). Cortical thickness differences in superior temporal gyrus have also been associated with suicide attempters (23) in SSD, consistent with the present study.

We further found decreased activation in the middle cingulate gyrus in the high vs. low SIB group. Previous work has established lower functional connectivity in subjects with suicidal ideation in the right posterior cingulate gyrus region, including both middle cingulate gyri, left transverse temporal gyrus, right supramarginal gyrus, left inferior parietal gyrus, and right superior temporal gyrus (65). These regions have been identified as part of a network involved in theory of mind and memory retrieval, and further associated with suicidal ideation when functional connectivity is impaired (65). Although no study has yet investigated the causal relationship between dysfunction in this network and suicidality, there is significant evidence for this network’s relationship to SIB, independent of MDD severity (65). Their finding for the right superior temporal gyrus is consistent with our finding that low activation in this region was associated with higher levels of urgency. It is also consistent with cortical thickness findings by Besteher et al. (23) in SSD. The presence of deactivation within the high SIB group, along with comparable unpleasantness ratings between groups, clarifies that this group was engaged in the task.

Negative urgency has been previously conceptualized as a loss of impulse control due to emotion regulation deficits (66). Neuroimaging studies of negative urgency have additionally related smaller cortical thickness in the dorsomedial PFC as well as the right temporal pole to urgency, emphasizing the neurobiological link between urgency and emotion regulation deficits (15, 18). Consistent with this idea, we previously published on the present sample, showing elevated urgency in people with higher SIB vs. lower levels of SIB in SSD (8).

Although differences in regions like the ACC are implicated in SSDs broadly, these effects cannot be attributed to the disorder itself. Rather, this study suggests that other factors, such as the compounding effects of emotion regulation defects and/or increased negative urgency contribute to heightened SI. One possibility noted earlier is that urgency is an outcome of poor emotion regulation (e.g., (66). Future studies comparing these findings in SSD to SI in other psychiatric disorders could help determine the diagnostic specificity of our fMRI results.

The findings in the current study suggest that hypoactivation in regions of the brain primarily responsible for emotion regulation and cognitive control may be associated with and contribute to increased suicidality. Moreover, activation in several of these regions is correlated with higher levels of urgency. In a study comparing siblings with SSD to healthy siblings, findings suggest impaired emotion regulation in SSD, accompanied by activation in the DLPFC, medial PFC, ACC, and amygdala (67). Hypoactivity within these regions has been associated with compromised cognitive control and emotion regulation within SSD patients (67). This emotion regulation deficit and its relationship to urgency may be a key mechanism leading to increased suicidality within this population.

Urgency has been shown to be elevated in a number of populations (11). In addition, others have found that elevated negative urgency is elevated in people with bipolar disorder and ideation, in one study (68), and attempts in another study (69). The effects in these studies were larger for negative than positive urgency, possibly highlighting the importance of intolerance of negative emotions in SIB transdiagnostically.

### Limitations

This study has several limitations. First, the sample size is small; it will be important to replicate these findings in a larger sample. For this reason, we were not able to test within-group correlations between brain activation and urgency. Additionally, we did not have a healthy or psychiatric control group (e.g., MDD); the specificity of our findings to SSD will need to be examined in a direct comparison. Furthermore, time since most recent suicide attempt was highly variable but was at least 6 months across participants. It will be of interest to study individuals with more recent attempts to examine emergent suicidal ideation. We also lacked power to test effects of racial group (70), sex (71) and lacked information on sexual identity (72), which are risk factors for SIB. The participants were chronically ill and were all on antipsychotic medication. It will be important to study SIB in first episode SSD, as this is a particularly high-risk population for SIB (73, 74). Finally, although activation in 3 extracted regions of group differences showed significant negative correlations with negative urgency, the voxelwise analysis of this relationship, which avoids issues of “double dipping” [e.g., (75)] was inconclusive, possibly due to low statistical power in this analysis.

## Conclusions

SIB in SSD has been under-studied. Here, we showed that during an emotion regulation task involving implicit reappraisal of negative pictures, activation was lower in the high SIB than low SIB group in several frontotemporal regions. These regions have been associated with emotion regulation processes, but interestingly, much of this activation was associated with lower levels of urgency. The deficit of activation in the high SIB group implies that the neural circuitry supporting emotion regulation is impaired and implicates this process in SIB. Moreover, the relationship between urgency and activation on the task suggests neural substrates for elevated urgency, as we previously reported (15). These results suggest important behavioral and brain targets for interventions that can be aimed at reducing SIB in schizophrenia.

## Data availability statement

The raw data supporting the conclusions of this article will be made available by the authors, without undue reservation.

## Ethics statement

The studies involving humans were approved by Nathan S. Kline Institute for Psychiatric Research Institutional Review Board. The studies were conducted in accordance with the local legislation and institutional requirements. The participants provided their written informed consent to participate in this study.

## Author contributions

MH: Conceptualization, Formal analysis, Funding acquisition, Investigation, Methodology, Project administration, Supervision, Visualization, Writing – original draft, Writing – review & editing. KE: Data curation, Investigation, Project administration, Writing – review & editing, Writing – original draft. ZP: Data curation, Investigation, Project administration, Writing – review & editing. AS: Writing – review & editing, Data curation, Investigation, Project administration, Supervision, Validation. ES: Writing – review & editing, Data curation, Formal analysis, Investigation, Project administration, Resources, Validation. AA: Conceptualization, Methodology, Writing – review & editing. DI: Conceptualization, Supervision, Writing – review & editing.
