# Supplementary material for: Emotion-related impulsivity and suicidal ideation and behavior in schizophrenia spectrum disorder: a pilot fMRI study
Source: Front Psychiatry. 2024 Jun 26;15:1408083. doi: 10.3389/fpsyt.2024.1408083 (PMC11234166; doi:10.3389/fpsyt.2024.1408083)
Supplement: Supplementary file 1 [file DataSheet_1.docx]

Emotion-related Impulsivity and Suicidal Ideation and Behavior in Schizophrenia Spectrum Disorder: A Pilot fMRI study – Supplementary Material

Matthew J. Hoptman^1,2^, Kathryn T. Evans^1,2^, Zamfira Parincu^1^, Allison M. Sparpana^1,2^, Elizabeth F. Sullivan^1,2^, Anthony O. Ahmed^3^, and Dan V. Iosifescu^1,2^

**Figure Captions**

**Supplementary Figure 1.** For the high SIB group only, Neutral/Negative – Neutral/Neutral contrast superimposed on cortical surface maps. Surfaces shown are lateral (left column) and medial (right column) for left (LH) and right (RH) hemispheres.Images thresholded using p < .005, cluster size 9.

**Supplementary Figure 2.** For the low SIB group only, Neutral/Negative – Neutral/Neutral contrast superimposed on cortical surface maps. Surfaces shown are lateral (left column) and medial (right column) for left (LH) and right (RH) hemispheres.Images thresholded using p < .005, cluster size 9.

**Supplementary Figure 3.** Map of significant correlations between Negative Urgency and Neutral/Negative – Neutral/Neutral contrast superimposed on an MNI space anatomical template. Images thresholded using p < .005. Warm colors indicate positive correlations, dark colors indicate negative correlations. Outlined areas are signficant at a clusterwise threshold of 9 voxels. Other areas are significant at subthreshold levels, with the intensity of coloring indicating the deviation below significance (after (Taylor et al., 2023)). Top) Axial view (every 5^th^ slice), Bottom) Coronal view (every 7^th^ slice).

Reference

Taylor, P.A., Reynolds, R.C., Calhoun, V., Gonzalez-Castillo, J., Handwerker, D.A., Bandettini, P.A., Mejia, A.F., Chen, G., 2023. Highlight results, don’t hide them: Enhance interpretation, reduce biases and improve reproducibility. NeuroImage 274, 120138. https://doi.org/10.1016/j.neuroimage.2023.120138


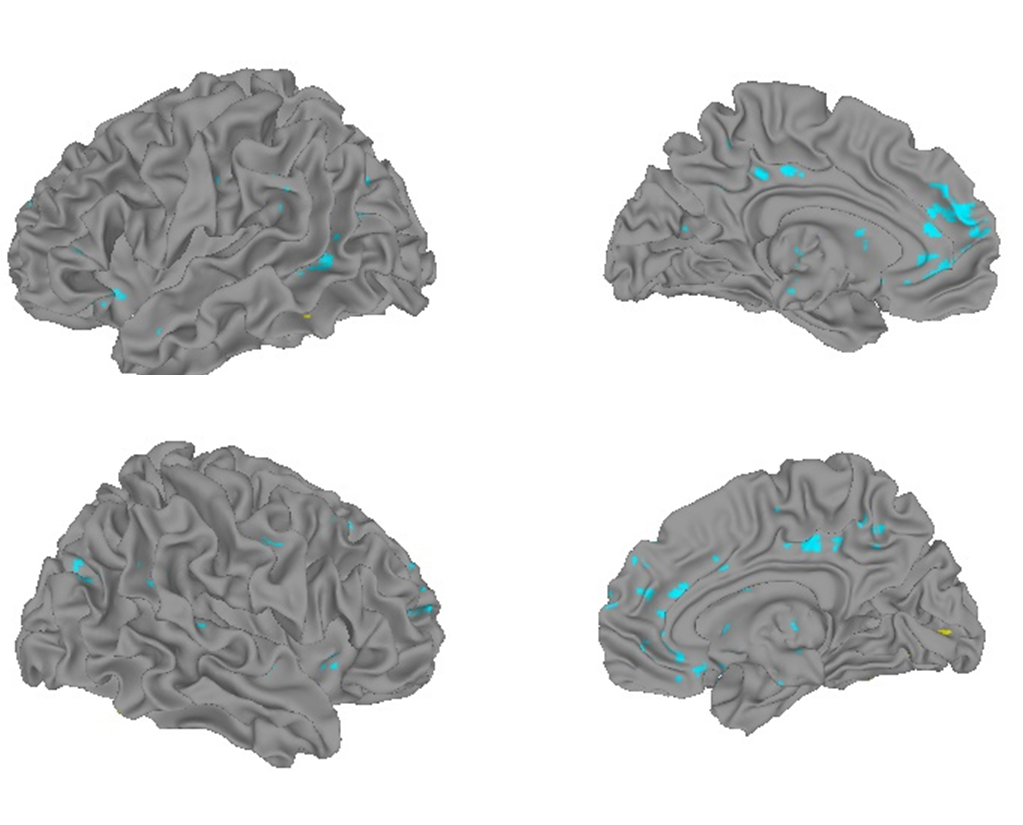


LH

RH

Lateral Surface

Medial Surface

**Supplementary Figure 1**

Lateral Surface

Medial Surface

LH

RH


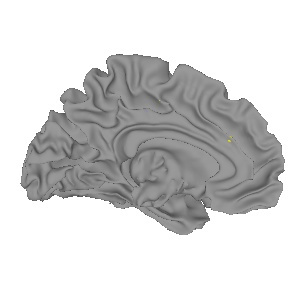

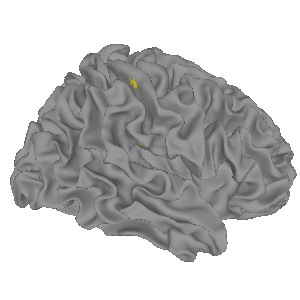

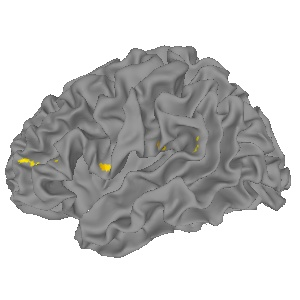

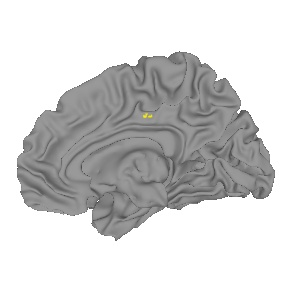


**Supplementary Figure 2**

**Supplementary Figure 3**

**
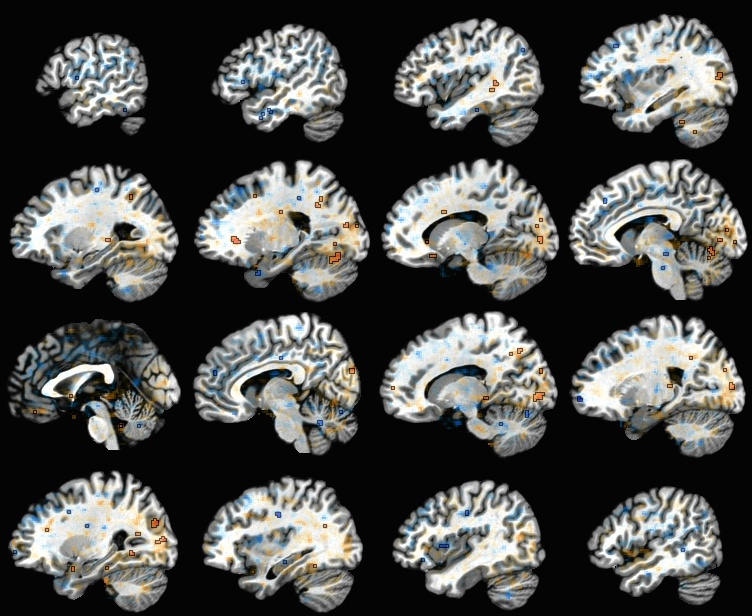
**

**
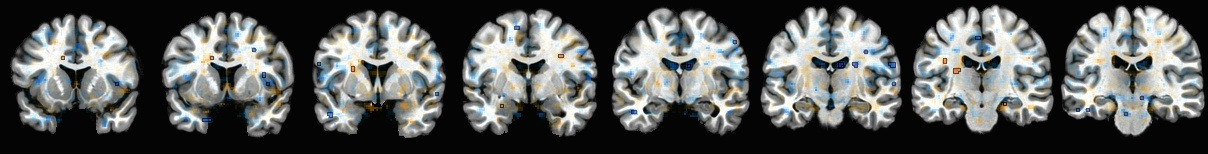
**
